# An ionic liquid- and PEO-based ternary polymer electrolyte for lithium metal batteries: an advanced processing solvent-free approach for solid electrolyte processing†

**DOI:** 10.1039/d3ra02488a

**Published:** 2023-06-14

**Authors:** Lukas Herbers, Verena Küpers, Martin Winter, Peter Bieker

**Affiliations:** a MEET Battery Research Center, Institute of Physical Chemistry, University of Münster 48149 Münster Germany; b Helmholtz-Institute Münster (HIMS) IEK-12, Forschungszentrum Jülich GmbH 48149 Münster Germany peter.bieker@uni-muenster.de

## Abstract

A processing solvent-free manufacturing process for cross-linked ternary solid polymer electrolytes (TSPEs) is presented. Ternary electrolytes (PEODA, Pyr_14_TFSI, LiTFSI) with high ionic conductivities of >1 mS cm^−1^ are obtained. It is shown that an increased LiTFSI content in the formulation (10 wt% to 30 wt%) decreases the risk of short-circuits by HSAL significantly. The practical areal capacity increases by more than a factor of 20 from 0.42 mA h cm^−2^ to 8.80 mA h cm^−2^ before a short-circuit occurs. With increasing Pyr_14_TFSI content, the temperature dependency of the ionic conductivity changes from Vogel–Fulcher–Tammann to Arrhenius behavior, leading to activation energies for the ion conduction of 0.23 eV. In addition, high Coulombic efficiencies of 93% in Cu‖Li cells and limiting current densities of 0.46 mA cm^−2^ in Li‖Li cells were obtained. Due to a temperature stability of >300 °C the electrolyte guarantees high safety in a broad window of conditions. In LFP‖Li cells, a high discharge capacity of 150 mA h g^−1^ after 100 cycles at 60 °C was achieved.

## Abstract 2

A processing solvent-free manufacturing process for cross-linked ternary solid polymer electrolytes (TSPEs) is presented.

## Graphical abstract

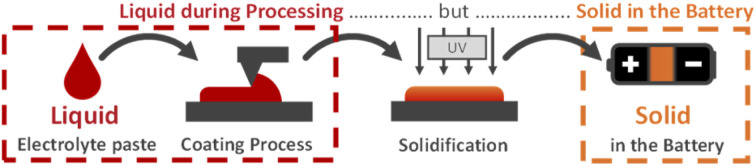

## Introduction

Lithium (Li) metal has become the focus of extensive research in the past years.^1^ Its high gravimetric capacity of 3860 mA h g^−1^ and low standard reduction potential of −3.04 V *vs.* standard hydrogen electrode (SHE) make it an ideal choice for a negative electrode active material in high energy density batteries.^2^ Nonetheless, the application of Li metal has major challenges.^3^ The decomposition of the electrolyte, the formation of the Solid Electrolyte Interphase (SEI) and the accumulation of inhomogeneous Li metal deposits, respectively ‘high surface area lithium (HSAL)’ and ‘dead Li’ cannot only result in active lithium loss (ALL) but also safety concerns.^4–6^ In particular, the risk of short-circuits by HSAL and the high reactivity of liquid flammable electrolytes with HSAL can be hazardous.^7,8^ To overcome these safety concerns, solid electrolytes (SE) and electrolytes based on ionic liquids are considered as safer alternatives.^9–11^

Solid electrolytes are often divided into the categories of inorganic solid electrolytes (ISEs), solid polymer electrolytes (SPEs) and composite polymer-inorganic electrolytes (CPIEs).^12^ The most studied polymer for Li-based batteries is poly ethylene oxide (PEO) due to its ether-functional groups combined with an alkyl-segment.^13^ The ether groups interact with Li-ions, granting a great Li salt solubility as well as good ion transport properties.^13^ Despite the benefit in safety of PEO electrolytes, they usually suffer from a low room temperature (RT) ionic conductivity.^14^ In comparison, several ISEs provide sufficient ionic conductivity. Nonetheless, ISEs can have challenges on their own, for instance a narrow electrochemical stability window, an expensive synthesis or poor electrode|electrolyte compatibility due to a low mechanical flexibility.^15,16^ Therefore, hybrids of different types of electrolytes are useful to balance the requirements of processability, ionic conductivity, safety and reversibility of Li metal electrodeposition and -dissolution.^17,18^ ISEs, SPEs and/or liquid electrolytes can therefore be combined to form CPIEs, gel and pseudo-solid electrolytes. The combination of polymers with RT ionic liquids (IL) and Li salts results in so called ternary solid polymer electrolytes (TSPE), with significantly higher ionic conductivities compared to SPEs and greater mechanical flexibility than ISEs.^19,20^ Moreover, while more expensive than organic solvent-based electrolytes, the low vapor pressure and high thermal stability of ionic liquids increases the safety compared to liquid organic electrolytes.^21^

Nonetheless, despite the higher performance by improved ionic conductivity compared to SPEs, TSPEs also show major challenges which need to be addressed to make them applicable in Li metal full cells. As shown by Zhang *et al.* a PEO-based TSPE (PEO_ref_) with a molar ratio of 10 : 1 : 2 (EO : LiTFSI (lithium bis(trifluoromethanesulfonyl)imide) : Pyr_14_TFSI (1-butyl-1-methyl-pyrrolidinium bis(trifluoromethylsulfonyl)imide)) suffers from low mechanical stability (elastic modulus: 0.3 MPa at 20 °C).^20,22^ This low mechanical strength results in a limited suppression of Li metal HSAL, which penetrates through the electrolyte leading to a cell short-circuit. In this work the ratio of polymer, Li salt and IL is balanced to benefit from the high ionic conductivity provided by the IL but at the same time enable short-circuit prevention by the high mechanical strength of Li salt and polymer. Furthermore, different from commonly used electrolyte processing by use of processing solvent-based casting or hot pressing of solids, which is expensive, time-consuming and in some cases even hazardous, in this work, only liquid or soluble precursors without any processing solvents are used. For this purpose, long chain solid PEO is replaced by PEODA (polyethylene oxide diacrylate), a small chain and therefore liquid SPE. Thus, the energy-consuming removal of the solvents during processing of solid CPIEs and TSPEs after solvent casting is not necessary. Also, in contrast to hot pressing, no elevated temperatures are needed, because the electrolyte can be processed under RT, making the presented electrolyte processing more time- and energy-efficient. During processing, the acrylate end groups of the PEODA are cross-linked by ultraviolet (UV) light induced radical cross-linking process, which leads to a fast phase transition from liquid to solid, enabling the combination of the processability of a liquid with the safety of a solid electrolyte. The ternary electrolyte composition is optimized towards a high conductivity and to meet the safety concerns regarding inhomogeneous Li electrodeposition and -dissolution, temperature stability and high voltage stability. Finally, the Li metal electrodeposition/-dissolution behavior in Li‖Li, Cu‖Li and lithium iron phosphate (LFP)‖Li cells and the electrochemical performance and limitations of the electrolyte in symmetric Li‖Li cells are determined.

## Experimental

### Chemicals

PEODA (Sigma-Aldrich, purity ≤100%, *M*_n_ = 700) was stored over activated molecular sieve 3 Å for at least seven days before use (water content: 20 ± 3 ppm by Karl-Fischer titration). Benzophenone (BP) (Merck, purity = 99%) was used as received. Pyr_14_TFSI (Solvionic, purity = 99.9%) was dried for 48 hours at 110 °C under vacuum followed by 48 hours at 110 °C under high-vacuum <10^−7^ mbar. LiTFSI (TCI, purity > 98%) was dried for at least 48 hours at 110 °C under vacuum followed by 48 hours at 110 °C under high-vacuum <10^−7^ mbar. LFP sheets (LiFePO_4_, NANOMYTE® BE-60E, experimental capacity ≥170 mA h g^−1^, NEI Corporation) were dried for 48 hours at 100 °C under vacuum.

### Preparation of electrolyte films

Electrolyte films were prepared in a dry room. The electrolyte “P_*x*_IL_*y*_S_*z*_” was named according to its mass ratios of *x* PEODA “P_*x*_”, *y* of the Pyr_14_TFSI “IL_*y*_” and *z* of LiTFSI “S_*z*_”, see Table 1. Different *x*, *y*, *z* mass ratios were mixed with BP (1 wt% of the polymer content). Afterwards, the liquid mixture was filled into a polytetrafluoroethylene (PTFE) mold with two different mold heights of 250 ± 25 μm or 500 ± 50 μm and covered by a siliconized biaxial-oriented polyethylene terephthalate (boPET) foil. The electrolyte was placed in an UVACUBE 100 with a 100 W lamp (Dr Höhnle AG) and was irradiated by UV light for 10 min. The final electrolyte thicknesses were validated by a layer thickness gauge (Mitutoyo ABSOLUTE) after cross-linking. PEO_ref_ was prepared as described elsewhere.^20,22^

**Table 1:** Abbreviation for P_*x*_IL_*y*_S_*z*_

Abbreviation	Component	Chemical
P	Polymer	PEODA
IL	Ionic liquid	Pyr_14_TFSI
S	Salt	LiTFSI

The 851 Titrando Karl Fischer Coulometer (Metrohm, Herisau, Switzerland) was calibrated with a threefold measurement of a 100 mg L^−1^ water standard for quantification. Each electrolyte was measured three times with an injection of 1 g. Instrument control, data acquisition and data evaluation were performed using tiamo™ 2.4 (Metrohm).^23^

The temperature stabilities of the electrolytes were studied using thermogravimetric analysis (TGA, A Q5000IR by TA instruments) with a heat rate of 10 K min^−1^ from 20 °C to 600 °C.

Fourier-transform infrared spectroscopy (FT-IR) measurements were performed on a BRUKER ALPHA II. The samples were placed onto the attenuated total reflection (ATR) crystal and a wavenumber range from 4000 cm^−1^ to 400 cm^−1^ was measured.

For electrochemical measurements round electrolyte discs of 15 mm diameter and 250 μm thickness were assembled in Li‖Li, Cu‖Li, stainless steel (SST)‖Li, LFP‖Li and SST‖SST CR2032 two-electrode coin cells in a dry room. Temperature dependent ionic conductivity profiles were measured by impedance spectroscopy in SST‖SST cells on a Novocontrol Alpha Analyzer. An amplitude of 10 mV, a frequency range from 0.1 Hz to 10 MHz and a temperature interval from 0 °C to 80 °C in 10 °C steps were applied. Galvanostatic polarization tests (0.1 mA cm^−2^, 0.5 mA h cm^−2^, 50 cycles) with Li‖Li cells, galvanostatic discharge tests (‚short-circuit tests‘, 0.1 mA cm^−2^) with Li‖Li cells, galvanostatic polarization tests (0.05 mA cm^−2^, 100 cycles, 2.5 V to 4.0 V) with LFP‖Li cells and galvanostatic polarization tests (0.1 mA cm^−2^, 0.1 mA h cm^−2^, 200 cycles, cutoff voltage ± 1 V) with Cu‖Li cells were performed on a MACCOR battery cycler (MACCOR Series 4000) at 60 °C. Linear sweep voltammetry tests (0.5 mV s^−1^, from OCV (open-circuit voltage) to 6 V) in SST‖Li cells, charge pulse analysis tests (1.2 mA cm^−2^, 1.4 mA cm^−2^, 1.6 mA cm^−2^, 1.8 mA cm^−2^, 2.0 mA cm^−2^ and 2.2 mA cm^−2^, cutoff voltage 1 V, rest time between charge pulses 3 h) in Li‖Li cells and transference number measurements (10 mHz to 1 MHz, ± 10 mV, 20 ± 1 mV) in Li‖Li cells were performed on a VMP potentiostat (Bio-152 Logic) at 60 °C. A triple measurement set was performed for each technique.

## Results and discussion

### Processability

**Fig. 1:**
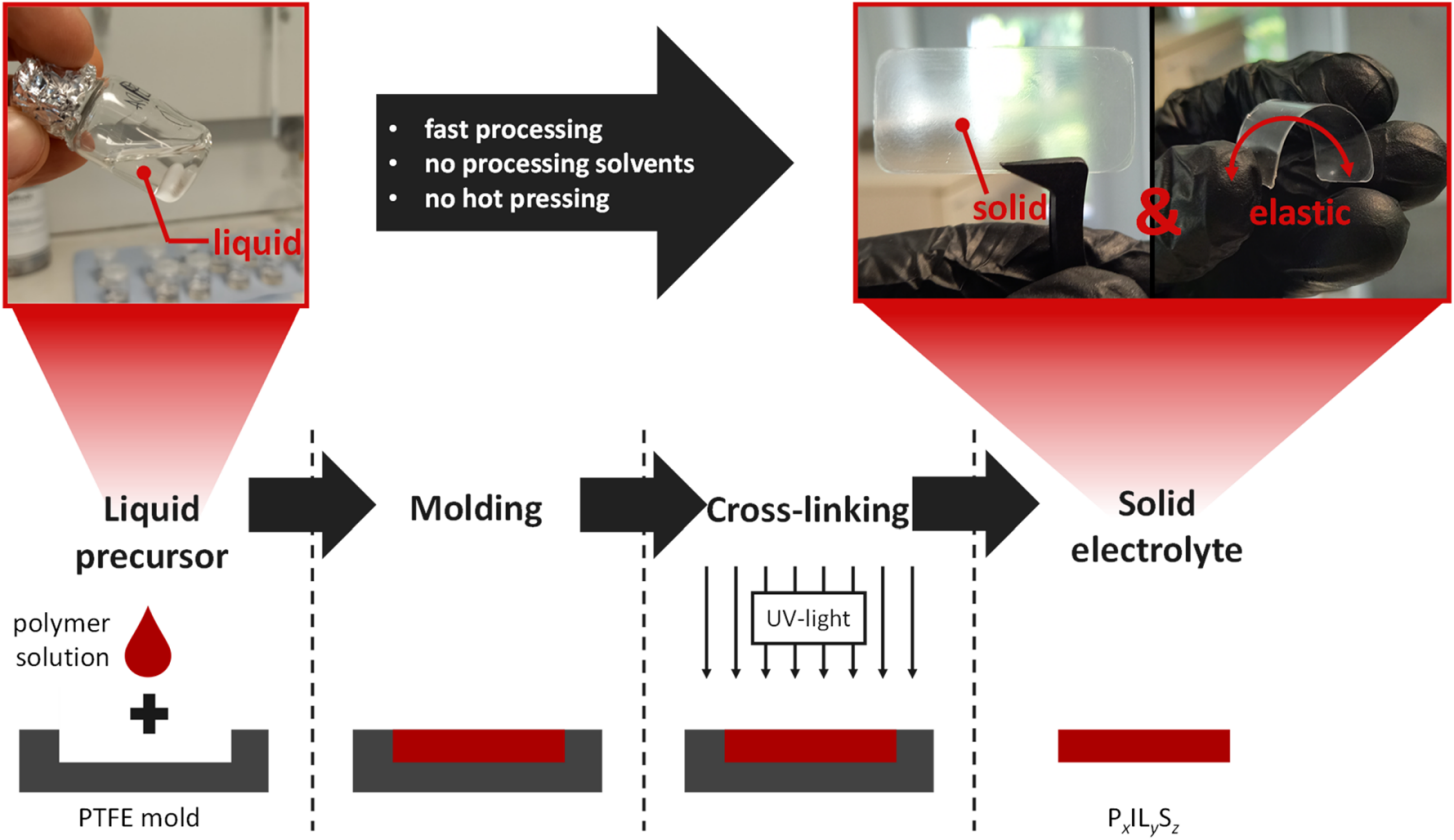
Processing solvent-free preparation ternary electrolyte manufacturing (liquid precursor, molding, cross-linking, solid electrolyte).

The processing of electrolytes of different composition is schematically given in Fig. 1. The mixture of precursors PEODA, Pyr_14_TFSI and LiTFSI results in a homogeneous liquid phase. PEODA is the key component enabling the fully liquid precursor as well as the solid final state of the electrolyte. Due to its small chain length, it is liquid at RT, making it a solvent-like component for LiTFSI. The ethylene oxide groups strongly interact with Li^+^ ions and dissolve LiTFSI even at high weight ratios of 40% in contrast to pure Pyr_14_TFSI-LiTFSI mixtures, which solidify at high LiTFSI contents. While the inner molecular chains (–CH_2_–CH_2_–O–)_*x*_ are beneficial for the liquid state, the end groups (CH_2_=CH–CO–O–R) of PEODA can lead to a phase formation of the electrolyte from liquid to solid. The acrylate groups at the end of the polymer chains contain double bonds that are cross-linked by radical induced polymerization.^24^ The full cross-linking mechanism is given in Fig. 11 in the ESI.†

**Fig. 2:**
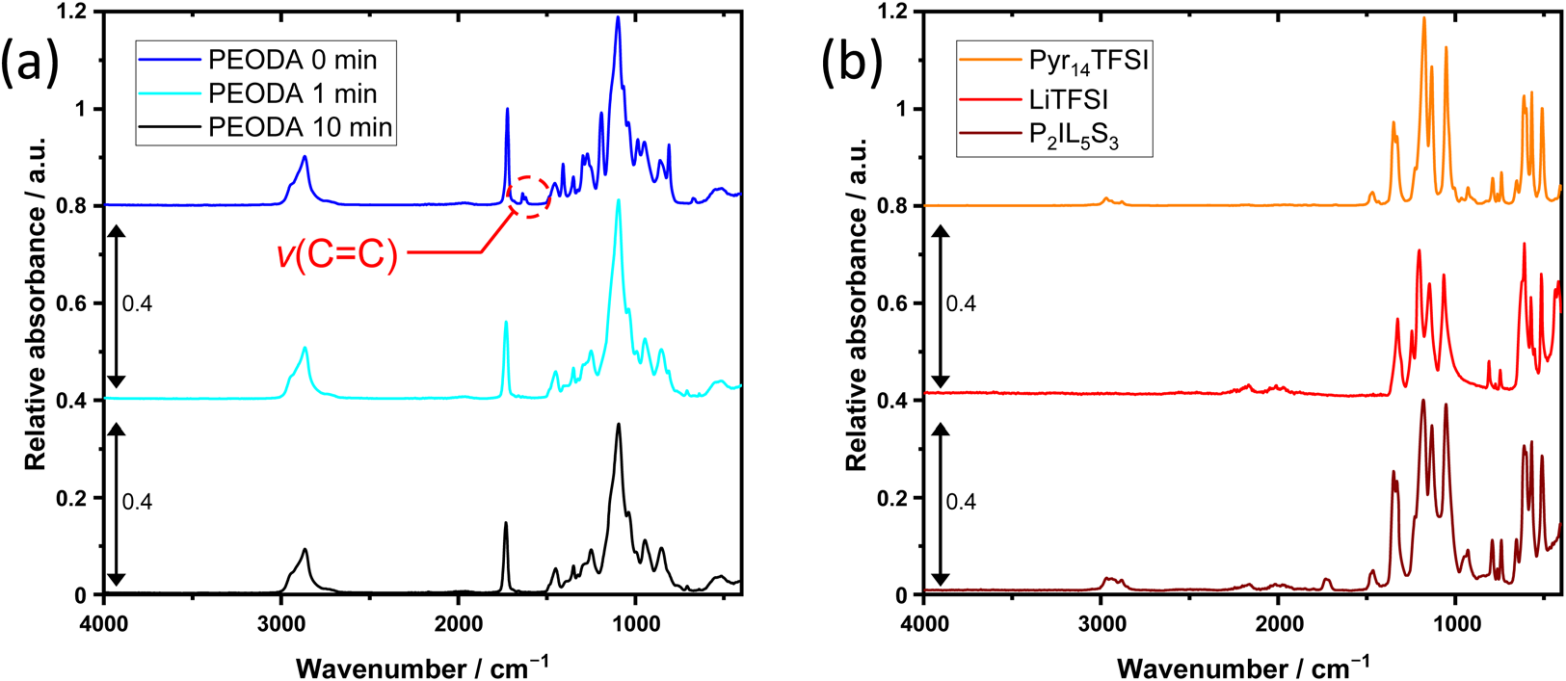
Absorbance spectra of (a) Pyr_14_TFSI, LiTFSI and P_2_IL_5_S_3_ cross-linked for 10 min and (b) PEODA with 1 wt% of BP not cross-linked, cross-linked for 1 min and cross-linked for 10 min.

The cross-linking process is verified by FT-IR analysis. As shown in Fig. 2a, the non-cross-linked PEODA (blue curve) has a band in the absorption spectra at a wavenumber of 1636 cm^−1^, which is caused by the valence vibration of the carbon–carbon double bond (*v*(C=C)). With increasing progress of the radical chain reaction, the peaks representing double bonds decrease, allowing to monitor the determination of the cross-linking process. As is seen from Fig. 2a no *v*(C=C) peak is observed even after 1 min of irradiation (cyan curve), indicating that despite the low weight percentage of photo initiator, the cross-linking is completed after 1 min, compare Fig. 12 in the ESI.† After 10 min of exposure to UV radiation (black curve) no further changes in the IR spectra are observed. To guarantee a fully cross-linked state even at lower polymer content, all electrolytes were cross-linked for an excessive time of 10 min. A spectra of the electrolyte P_2_IL_5_S_3_ (brown curve) after 10 min of UV radiation, as well as the spectra of the pure Pyr_14_TFSI (orange curve) and pure LiTFSI (red curve) used for electrolyte mixing, are shown in Fig. 2b. No *v*(C=C) peak is observed in the P_2_IL_5_S_3_ spectra. The resulting films are transparent, homogeneous, non-porous and elastic (elastic modulus: 3.2 MPa). (see Fig. 1 and 13 in the ESI†).

### Ionic conductivity

**Fig. 3:**
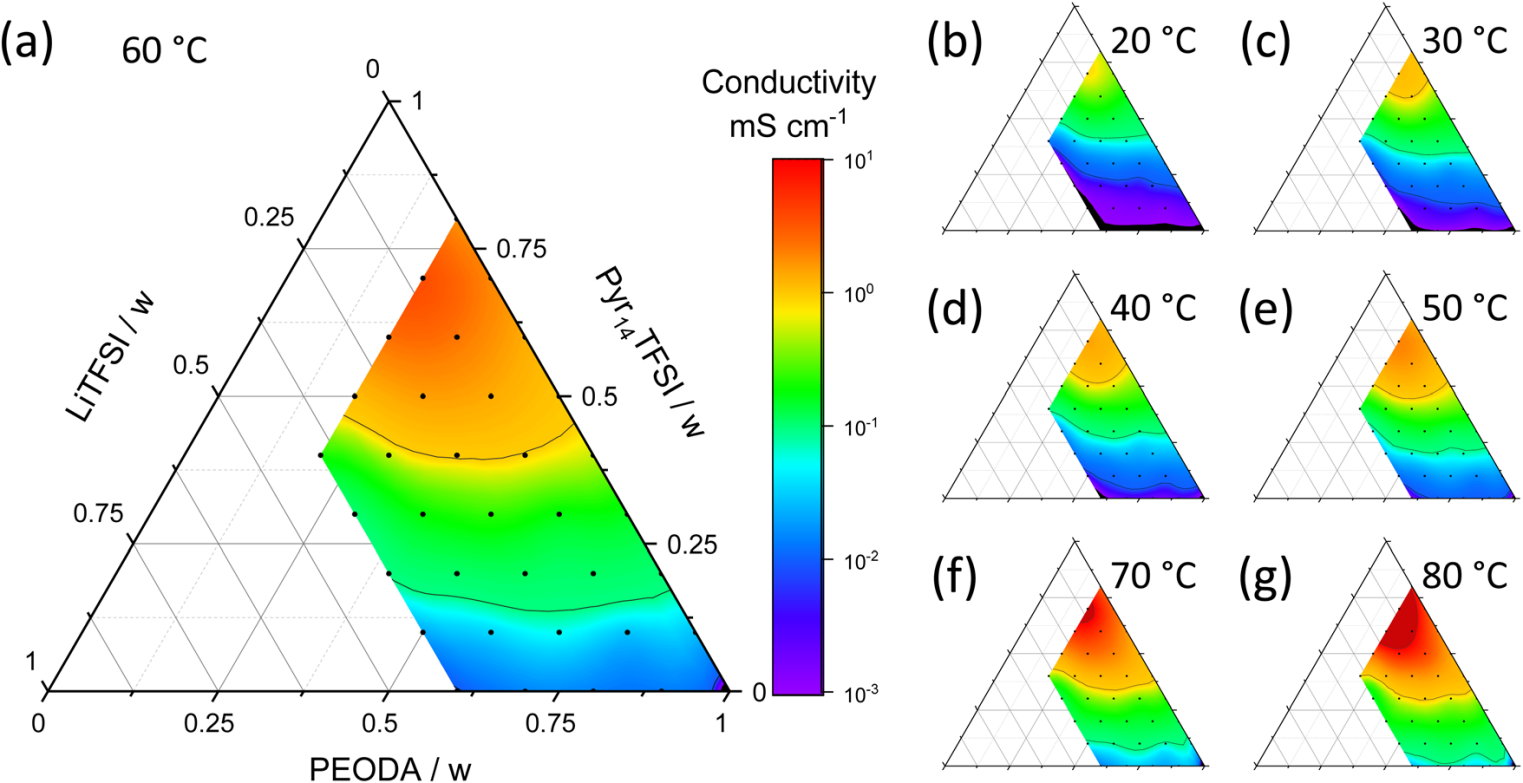
Ionic conductivities of different electrolyte compositions at various temperatures. Ternary ionic conductivity diagram at (a) 60 °C; (b) 20 °C; (c) 30 °C; (d) 40 °C; (e) 50 °C; (f) 70 °C; (g) 80 °C. Black dots indicate a measured sample, ionic conductivities <10^−3^ mS cm^−1^ are marked in black and ionic conductivities of >10 mS cm^−1^ are dark red regions.

The ternary diagrams of various mass ratios of Pyr_14_TFSI, LiTFSI and PEODA at different temperatures are shown in Fig. 3. The ionic conductivities from 10^−3^ mS cm^−1^ (purple) to 10^1^ mS cm^−1^ (red) are plotted over the composition in weight fraction. Seven temperatures from 20 °C to 80 °C are shown. Due to viscosity limitations the weight fraction of LiTFSI is restricted to a maximum of 0.4. The polymer content is kept between 0.2 and 1.0 to enable sufficient mechanical stability of the electrolyte.

An ionic conductivity of ≥1 mS cm^−1^ is considered as a benchmark in literature.^17,25^ This benchmark is not reached for a temperature of 20 °C independent of the mass ratios of the ternary electrolyte, see Fig. 3b, but at slightly elevated temperature of 30 °C (Fig. 3c) for high Pyr_14_TFSI fractions. An ionic conductivity of ≥1 mS cm^−1^ is achieved for smaller fractions of Pyr_14_TFSI when increasing the temperature from 40 °C to 60 °C (Fig. 3a, d and e). When increasing the temperature even further to 70 °C and 80 °C, ionic conductivities of 10 mS cm^−1^ and higher are reached, Fig. 3f and g.

In order to provide a high ionic conductivity for a broad variety of mass ratios, a temperature of 60 °C (Fig. 3a) is chosen for further analysis. The conductivity range at this temperature differs by several orders of magnitude. In order to understand this behavior, the ionic conductivity *σ* of an electrolyte mixture can be viewed as a function of number density *n*, charge *q* and mobility *μ* of ions, see eqn (1).^13^1
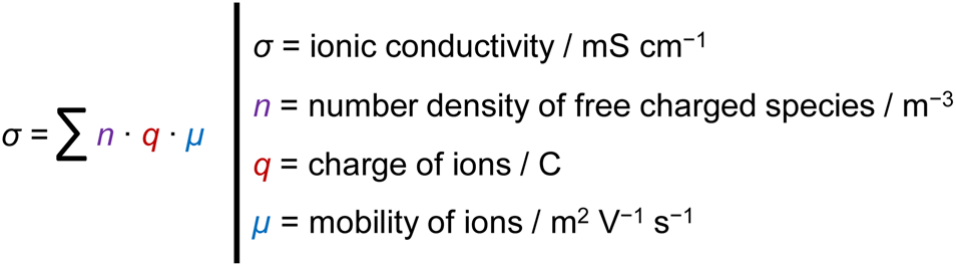


The highest conductivities are achieved with high Pyr_14_TFSI content. Samples with a Pyr_14_TFSI content of ≥50 wt% show conductivities greater than 1 mS cm^−1^, while electrolyte samples with high PEODA as well as high LiTFSI content show comparatively low conductivities. Pyr_14_TFSI is very ion conductive because the liquid state of the molten salt results in a high mobility *μ* of charge carriers.^26^ Exchanging PEODA by Pyr_14_TFSI elevates the number density *n* and the overall amount of charges because the concentration of single charge ions like Pyr_14_^+^ and TFSI^−^ is increased. In comparison to Pyr_14_TFSI and LiTFSI, PEODA is non-ionic and therefore decreases the number density of free charged species *n* and the charge *q* when increased. Furthermore, when PEODA is cross-linked, it turns from a liquid into a solid. This additionally decreases the ionic conductivity by reducing the overall mobility *μ*. To achieve conductivities as high as possible, PEODA is therefore reduced in amount to a minimum of 20 wt%. Nonetheless, PEODA is necessary because it is the key component enabling a liquid state as well as the solvent-free phase transition from liquid to solid as described earlier. An increased LiTFSI content improves the ion conductivity only to some extent. While the interaction of ethylene oxide from PEODA and Li^+^ promotes the LiTFSI dissociation and thereby the number density of free charged species *n*, the formation of ion clusters at high LiTFSI concentrations decreases the ionic conductivity.^27,28^ Thus [Li(TFSI)_*n*_]^(*n*−1)−^-complexes are formed, leading to a lower mobility *μ*.^29^ These effects lead to an optimal low LiTFSI content of 10 wt%. With a PEODA content of 20 wt% the ionic conductivity is 6.8 mS cm^−1^ for sample P_2_IL_7_S_1_. When the LiTFSI content is increased, the ionic conductivity is decreased (P_2_IL_6_S_2_: 4.5 mS cm^−1^; P_2_IL_5_S_3_: 1.3 mS cm^−1^; P_2_IL_4_S_4_: 0.4 mS cm^−1^). In conclusion, low PEODA-, high Pyr_14_TFSI- and moderate LiTFSI-contents are optimal for high ionic conductivity.

**Fig. 4:**
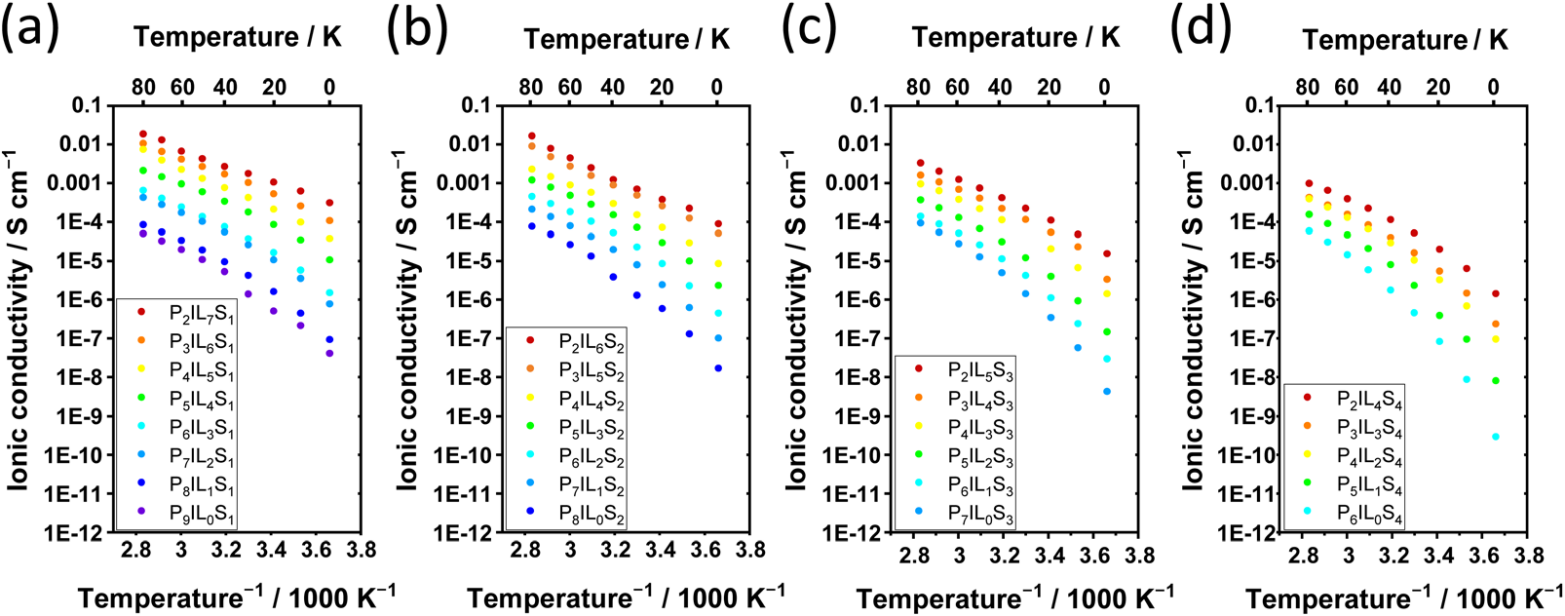
Temperature dependent ionic conductivities of ternary electrolytes with PEODA, Pyr_14_TFSI, and LiTFSI. Ionic conductivities plotted over inverse temperature and temperature; with LiTFSI compositions of (a) 10 wt%, (b) 20 wt%, (c) 30 wt%, and (d) 40 wt%.

The ionic conductivity behavior over the inverse of temperature can give insights into the phase behavior as well as the temperature dependency of ion conduction in electrolytes (Fig. 4).^13^

In Fig. 4a, the plots at high polymer ratios of ≥40 wt% show a non-linear curved trend of the ionic conductivity (logarithmic scaling) over the inverse temperature, which represents typical VFT (Vogel–Fulcher–Tammann) behavior.^13^ Therefore, the temperature dependent ionic conductivity is described by the VFT equation (eqn (2)). The VFT equation was originally postulated to describe the viscosity behavior of amorphous polymers above the glass transition temperature *T*_G_. Because the viscosity and ion mobility can depend on each other, the VFT equation is widely used in polymer electrolyte research to describe the temperature dependency of the ionic conductivity.^30^ The ion transport is described by the activation energy for ionic conduction *E*_A_, the ideal glass transition temperature *T*_0_ also known as Vogel temperature (usually about 50 K below *T*_G_), the Boltzmann constant *k*_B_ and the pre-exponential factor *σ*_0_.2
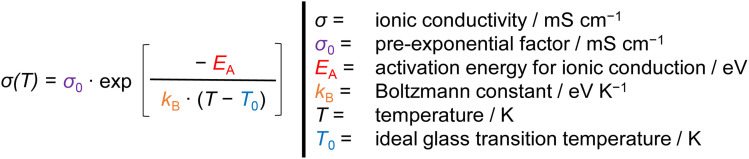


As shown from Fig. 4a–d, this behavior is valid for all compositions with a solid content (PEODA and LiTFSI) ≥50 wt%. The characteristic bending of the logarithmic scaled conductivity over the inverse temperature is caused by the reduction of the denominator *T* by *T*_0_ in the exponent of eqn (2). In Fig. 4d a steeper slope is seen from P_2_IL_4_S_4_ to P_6_IL_0_S_4_. This highlights an increased temperature influence when the liquid content (Pyr_14_TFSI) is exchanged by PEODA. This is explained by the strong temperature dependency of the segmental motions in polymers, which facilitate ion conduction.^15^ Due to the VFT behavior, an amorphous state without phase transitions is assumed in the measured temperature range. This is verified by DSC *e.g.*, for P_2_IL_5_S_3_ (see ESI, Fig. 14†).

With increasing Pyr_14_TFSI and decreasing PEODA amounts, a transition from a VFT (sample P_9_IL_0_S_1_) to a linear behavior (sample P_2_IL_7_S_1_) occurs for LiTFSI shares of ≤30 wt% (Fig. 4a–c). The linearity of the logarithmically scaled ionic conductivity over the inverse temperature is observed when *T*_0_ ≪ *T*. The ionic conductivities of these electrolytes follow the Arrhenius equation in which *T*_0_ is not considered, see eqn (3).3
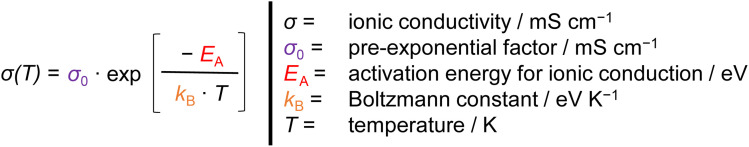


This behavior is typical for high Li salt or high ionic liquid content polymer electrolytes called PISE (polymer-in-salt electrolyte) or iongels.^13,31,32^ For P_2_IL_7_S_1_ (Fig. 4a, red) and P_2_IL_5_S_3_ (Fig. 4c, red) the activation energy *E*_A_ was derived by fitting the values according to the Arrhenius equation (see ESI, Fig. 15†). Two major differences are visible when comparing the Ionic conductivities plotted over inverse temperature for P_2_IL_7_S_1_ and P_2_IL_5_S_3_. First the values for P_2_IL_7_S_1_ show higher conductivities as a result of the higher ion mobility *μ* discussed previously, which is expressed by a higher *σ*_0_ in the Arrhenius equation. Second the slope of the ionic conductivities plotted over inverse temperature is lower for P_2_IL_7_S_1_ compared to P_2_IL_5_S_3_, which is expressed by a lower *E*_A_ in the Arrhenius equation. The activation energies are 0.18 eV and 0.23 eV for P_2_IL_7_S_1_ and P_2_IL_5_S_3_, respectively. The values are in agreement with comparable electrolytes from literature.^33^5
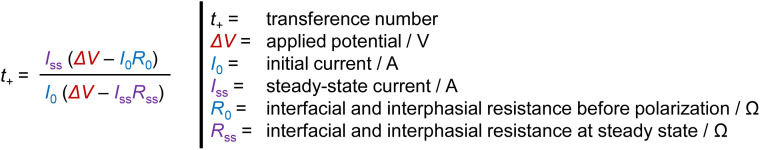


In summary, an Arrhenius type conductivity with low temperature dependency is achieved for electrolytes with high Pyr_14_TFSI portion. This behavior is favorable compared to VFT-like properties, in which the conductivity drops faster with decreasing temperature. Therefore, Li metal batteries containing electrolytes with high Pyr_14_TFSI content can be run in a wider temperature range.

### Safety and stability

**Fig. 5:**
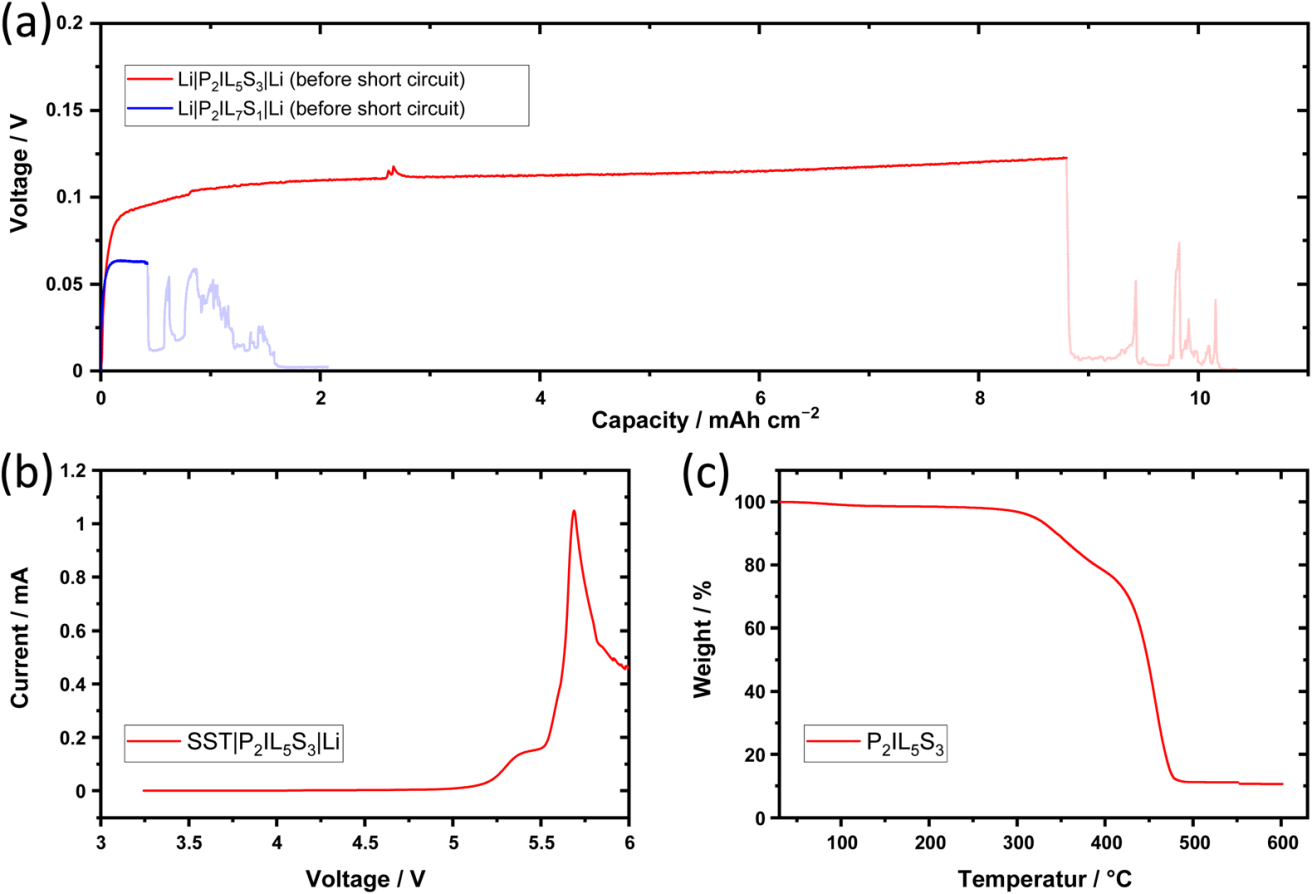
Safety & thermal stability of ternary solid electrolyte. (a) Li|P_2_IL_5_S_3_|Li cell - single discharge polarization of 0.1 mA cm^−2^ until failure at 60 °C; (b) LSV of a SST|P_2_IL_5_S_3_|Li cell from OCV to 6 V with a scan rate of 0.5 mV s^−1^ at 60 °C; (c) TGA of P_2_IL_5_S_3_ from 20 °C to 600 °C.

Apart from high ionic conductivity, electrolytes have to match the safety expectations for Li metal batteries. Especially the formation of HSAL, for example Li dendrite growth through the separator/electrolyte, cause a conductive connection of the electrodes and lead to a cell failure by short-circuit. To investigate the ability of the electrolytes of preventing short-circuits by dendrites single discharge polarization tests are performed, see Fig. 5. A rapid voltage drop indicates a short-circuit. For a symmetric Li‖Li cell with P_2_IL_7_S_1_, a short-circuit occurs after 0.42 mA h cm^−2^, which is too low to be used in practice. But for the Li‖Li cell using an electrolyte with increased LiTFSI content like P_2_IL_5_S_3_ a short-circuit occurs after deposition of 8.80 mA h cm^−2^. The capacity is increased by a factor of 20 making it applicable in practice. By partially exchanging Pyr_14_TFSI with LiTFSI, a trade off is made. On the one hand, an increased solid content (LiTFSI or PEODA) reduces the ionic conductivity as discussed above. On the other hand, a reduced liquid content (Pyr_14_TFSI) improves the mechanical stability of the electrolyte into a more rigid electrolyte, which reduce the risk of short-circuits caused by dendrites.^34^ The reason for increasing the solid content by LiTFSI instead by PEODA is discussed in the ESI (Fig. 16†).

The oxidative stability of the electrolyte P_2_IL_5_S_3_ is studied by linear sweep voltammetry with a sweep rate of 0.5 mV s^−1^ (OCV to 6 V) in a SST‖Li setup (Fig. 5b). A major current increase is observed for voltages >5.0 V. The current exceeds a value of 10 μA at 5.03 V and increases rapidly from >5.5 V. Therefore accoding to LSV the electrolyte is suitable to operate within the voltage range of common cathode materials like LFP with a average charge–discharge voltage of 3.4 V – 3.8 V *vs.* Li|Li^+^.^25,35,36^ Nonetheless, as shown by Jung *et al.* the applicability of an electrolyte towards a cathode material is not only determined by the high voltage stability of an electrolyte, but also by the chemistry of the cathode materials.^37,38^ As mentioned in their work additionally to the electrochemical oxidation pathway of the electrolyte, also the chemical oxidation has to be considered. Due to oxygen release of the NMC622 cathode material at high rates of discharge, which is excellerated at increased temperatures, the overall stability of the system can be limited to voltages <5 V *vs.* Li|Li^+^. Furthermore, as reviewed by Cabañero *et al.* the high voltage stability of the electrolyte is effected by the chemical reactivity of the cathode aswell as its surface area.^39^ In case of TSPEs the oxidation of PEO or PEODA at the cathode interface can limit the electrolyte stability and the application towards NMC622.^35,40,41^ Therefore, additionally to the LSV measurement, the effect of cycling towards LFP and NMC622 are shown in Fig. 10 and 19 in the ESI.† In order to apply NMC cathodes the TSPE composition has to be modified, which is of interest in an upcoming work.

The temperature stability of the electrolyte is measured by TGA over a temperature range from 20 °C to 600 °C. At ≥300 °C the decomposition of PEODA is observed.^42^ From 360 °C on, LiTFSI and Pyr_14_TFSI decompose.^42–44^ In summary, an overall high temperature stability of 300 °C is reached, which is comparable to PEO-Pyr_14_TFSI-LiTFSI-based electrolytes reported in literature.^44^

**Fig. 6:**
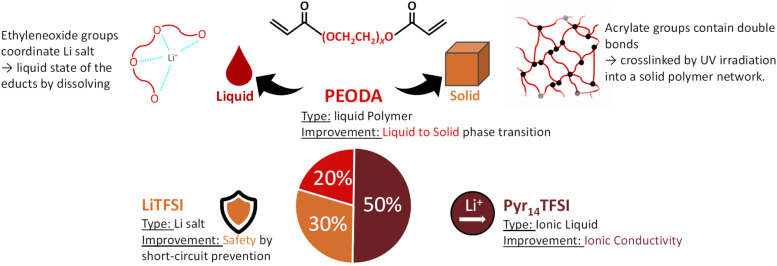
Summary of the main improvements provided by the ingredients of the P_2_IL_5_S_3_ electrolyte.

The optimized TSPE composition of P_2_IL_5_S_3_ is shown in Fig. 6. In summary, the 20 wt% of PEODA enable the liquid to solid phase transition, the 50 wt% of Pyr_14_TFSI boost the ionic conductivity compared to IL-free TSPEs and the 30 wt% LiTFSI improve the short-circuit prevention.

### Electrochemical performance

To reveal electrochemical performance and limitations of the electrolyte, the transference number *t*_+_ is measured by the Bruce–Vincent method.^45^ A small constant voltage (Δ*V*) of 20 mV ± 1 mV is applied to a Li|P_2_IL_5_L_3_|Li cell. The current drops from the initial current (*I*_0_) until a constant concentration gradient of ions over the electrolyte is achieved and a lower steady state current (*I*_SS_) is reached. In the ideal case, the steady state current *I*_SS_ is divided by the initial current *I*_0_ results in the *t*_+_, see eqn (4).4
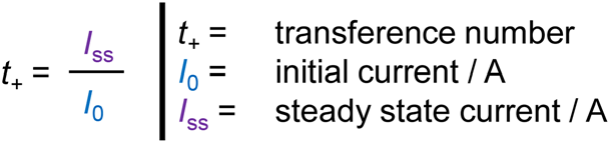


For solid electrolytes, overvoltages resulting from the interfacial/interphasial (I&I) resistance must be considered, as well. The extended equation with correction terms for the I&I resistance with the I&I resistance before polarization *R*_0_ and the I&I resistance under steady state *R*_SS_ is shown in eqn (5).

**Fig. 7:**
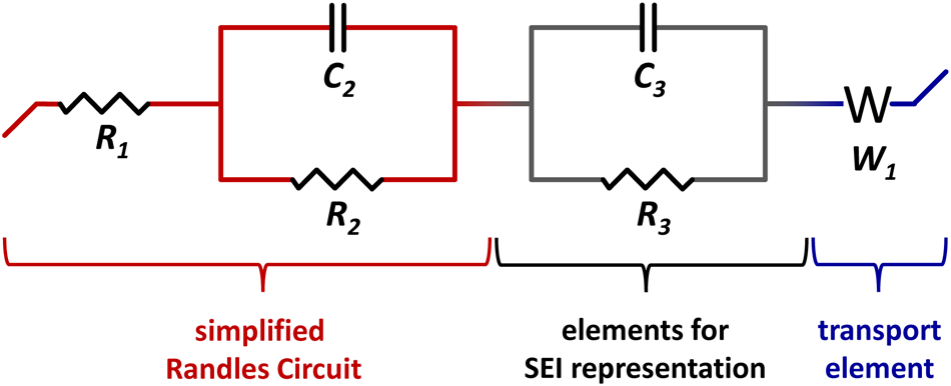
Equivalent circuit used to fit impedance plots.

**Fig. 8:**
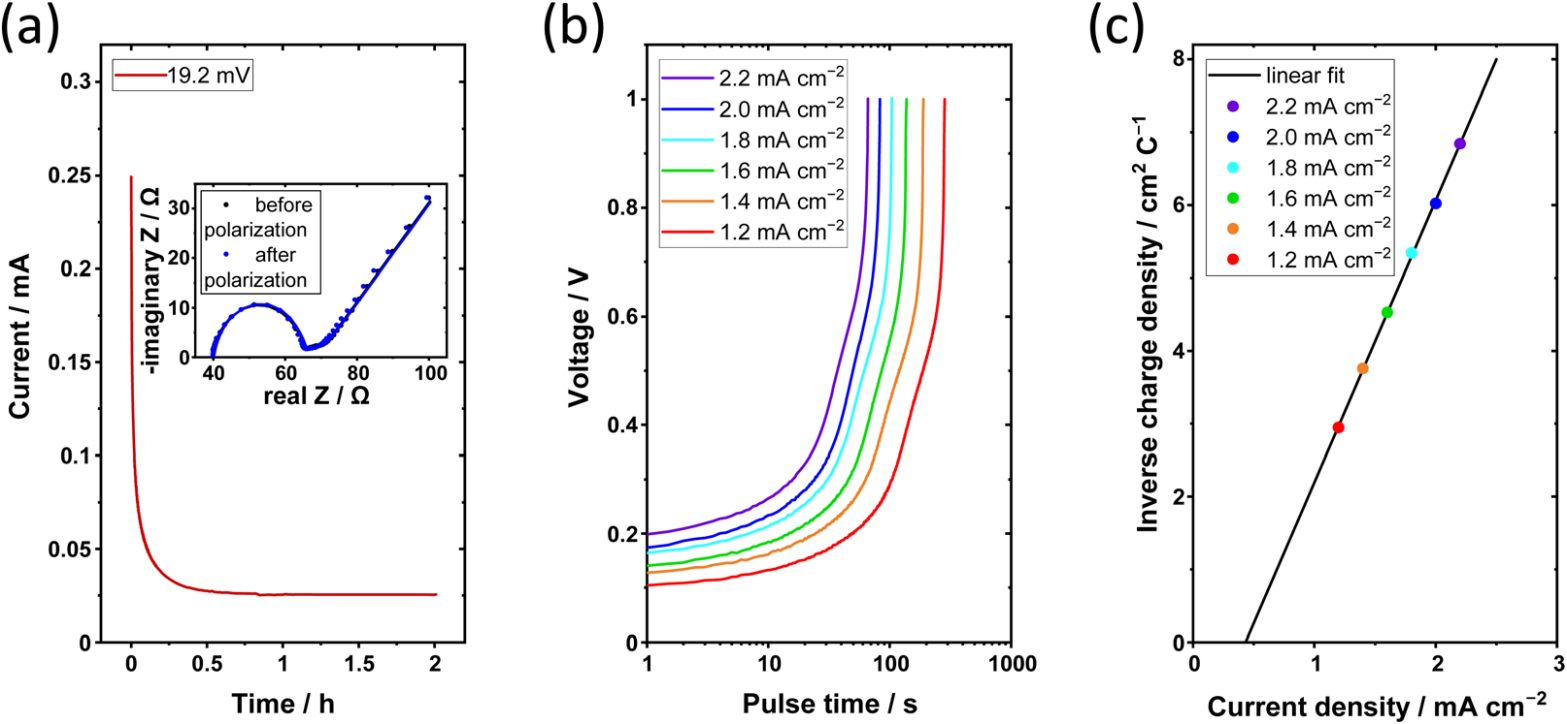
Electrochemical limitations of the ternary solid electrolyte. (a) Constant voltage polarization at 19.2 mV of a Li|P_2_IL_5_L_3_|Li cell at 60 °C, inset shows impedance measurement before and after polarization; (b) charge pulses from 1.2 mA cm^−2^ to 2.2 mA cm^−2^ of a Li|P_2_IL_5_L_3_|Li cell at 60 °C; (c) inverse charge density over current density, intersection of the linear fit with the current density axis represents the limiting current density.

I&I resistances are determined by electrochemical impedance spectroscopy. The impedance spectra are fitted by a representative circuit built of the elements: resistor, capacitor and transport (Warburg) element. The simplified Randles Circuit represents an ideal symmetric cell without diffusion and is extended to use it for fitting the Nyquist plots, see inset Fig. 8a. *R*_1_ is the electrolyte resistance. The parallel connection of the *R*_2_/*C*_2_ element represents the Li|electrolyte interface. *C*_2_ is the capacitive behavior of the ionic double layer at the Li|electrolyte interface and *R*_2_ represents the resistance for charge-transfer at the Li|electrolyte interface. A transport (Warburg) element (*W*_1_) for semi-infinite diffusion is used representing Li-ion diffusion at low frequencies. Because the *R*_1_ + (*R*_2_/*C*_2_) + *W*_1_ circuit does not take into account non-ideal phenomenons like the formation of an additional interphase by a SEI a second *R*_3_/*C*_3_ element is added.^46^ The equivalent circuit *R*_1_ + (*R*_2_/*C*_2_) + (*R*_3_/*C*_3_) + *W*_1_ is shown in Fig. 7.

The current response of a Li| P_2_IL_5_L_3_|Li cell at a constant voltage of 19.2 mV is shown in Fig. 8a (*I*_0_ = 0.2493 mA, *I*_SS_ = 0.0254 mA). To determine the *t*_+_ by the Bruce–Vincent method, impedance spectra are measured before and after polarization. The inset shows the Nyquist plots before and after polarization fitted by the equivalent circuit of Fig. 7 (*R*_0_ = 28.97 Ω, *R*_SS_ = 29.25 Ω). An average *t*_+_ of 0.063 ± 0.004 is determined. The *t*_+_ highlights the formation of [Li(TFSI)_*n*_]^(*n*−1)−^ complexes at high LiTFSI content, which, on one hand, decreases Li-ion mobility and reduces *t*_+_, but on the other hand, results in a great prevention of dendrite growth, as discussed before. The value is comparable to other Pyr_14_TFSI-based systems form literature with a *t*_+_ of 0.09.^47^

A charge-pulse method was applied to investigate the limiting current density.^48^ The resulting voltage over time plots for charge-pulses of 1.2 mA cm^−2^ to 2.2 mA cm^−2^ are shown in Fig. 8b. The cutoff voltage (1 V) is reached faster with increasing current density, indicating a faster depletion of Li^+^ in the electrolyte at the Li metal|electrolyte interface. As described by Wetjen *et al.*, the depletion time and the current density are used to derive the inverse charge density over current density, see Fig. 8c. By linear fitting the limiting current density is obtained from the *x*-axis intercept. The resulting limiting current density is 0.46 ± 0.03 mA cm^−2^. This high limiting current density highlights that despite the low *t*_+_ an electrolyte capable of cycling at sufficient rates has been synthesized.

### Reversibility of Li metal electrodeposition and -dissolution

**Fig. 9:**
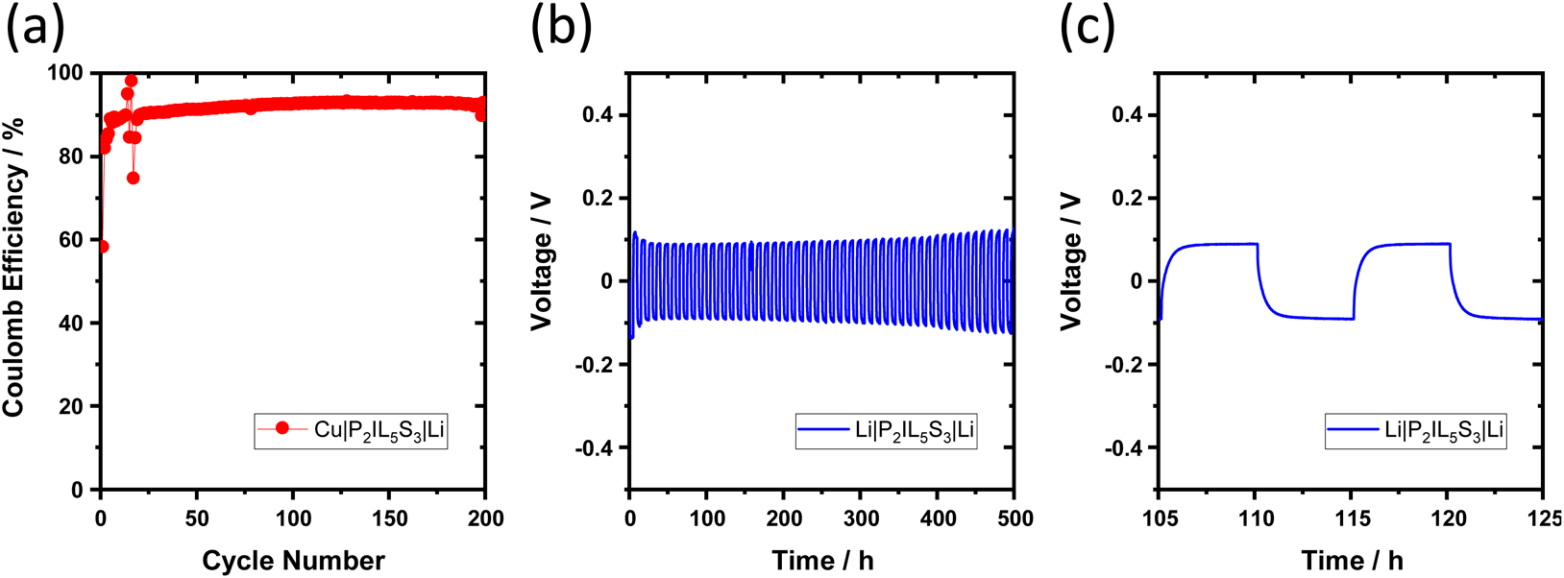
Li metal electrodeposition and -dissolution with a ternary solid electrolyte. Galvanostatic cycling of (a) Cu|P_2_IL_5_L_3_|Li cell at 0.1 mA h cm^−2^ for 0.1 mA cm^−2^ per step at 60 °C; (b) Li|P_2_IL_5_L_3_|Li cell at 0.1 mA cm^−2^ for 0.5 mA h cm^−2^ per step at 60 °C; (c) voltage profile of b from 105 h to 125 h.

The Coulombic efficiency of Li metal electrodeposition and -dissolution is studied by cycling a Cu‖Li cell.^49^ During electrodeposition/dissolution of Li metal on/from the Cu surface, active Li is lost by the formation of dead Li or side reactions causing a Coulombic efficiency <100% such as SEI formation. In a Cu‖Li cell with the electrolyte P_2_IL_5_S_3_, a Coulombic efficiency of 58% is determined in the first cycle (Fig. 9a). With increasing cycle number, the Coulombic efficiency increases to 93% where it stabilizes after the 90^th^ cycle. In comparison to PEO_ref_ from literature the Coulombic efficiency of P_2_IL_5_L_3_ in Cu‖Li cells is higher as well as more stable.^22^ The homogeneity of Li metal deposition is further evaluated by using a laser scanning microscope, see Fig. 18.†

The stability of Li cycling is studied in a Li‖Li cell. The voltage over time profile is shown in Fig. 9b. The high overvoltage in the first cycle (−0.14 V, 0.12 V) is reported to be caused by the native SEI of Li_2_O and Li_2_CO_3_ on the Li metal surface.^50^ After the first three cycles, the overvoltage drops to ±0.09 V due to the deposition of fresh Li metal without a thick SEI layer, as well as an presumably increased Li surface area.^8^ Because of the accumulation of dead Li, the Li-ion diffusion through the I&I of the Li metal anode becomes more resistive at later cycles leading to the overvoltage slightly increasing to ±0.12 V after 500 h.^51^

**Fig. 10:**
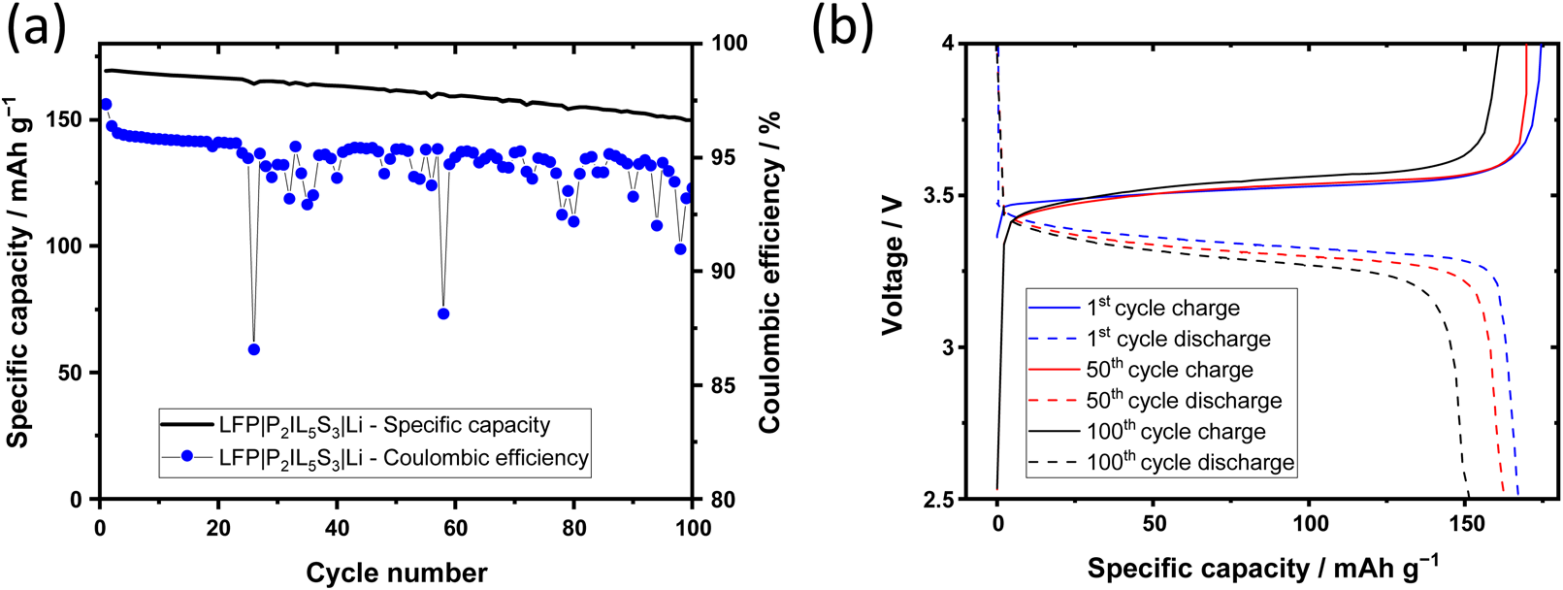
Li metal electrodeposition and –dissolution with a ternary solid electrolyte in LFP|P_2_IL_5_S_3_|Li cells at 0.05 mA cm^−2^ for 100 cycles at 60 °C. (a) Specific capacity and Coulombic efficiency over cycle number; (b) voltage evolution of the 1^st^, 50^th^, and 100^th^ cycle over the specific capacity.

Additionally, the stability of Li cycling as well as the Coulombic efficiency of Li metal electrodeposition and -dissolution is verified in LFP‖Li cells with P_2_IL_5_S_3_ electrolyte, see Fig. 10. Due to flexibility of the electrolyte the rough cathode surface is contacted well by the P_2_IL_5_L_3_ electrolyte, see Fig. 17 in the ESI.† The Coulombic efficiency of LFP|P2IL5S3|Li cells is 99% in the first cycle and drops to an average of 94% after 10 cycles at which it remains stable, see Fig. 10a. The Coulombic efficiency is slightly higher than in Cu‖Li cells (93%) which is possibly caused by a higher Li loss of highly reactive HSAL on the Cu surface. During cycling, the specific capacity remains at a high level, it starts at 169 mA h g^−1^ which is close to the maximum theoretical capacity of LFP. At the 100^th^, cycle 88% (150 mA h g^−1^) of the initial capacity is maintained, see Fig. 10a and b. During cycling, the overvoltage increases slightly which can be explained by the SEI and dead lithium formation discussed previously.

Finally, the P_2_IL_5_S_3_ is compared to a PEO-based electrolyte PEO_ref_ from literature, see Table 2.^20,22^ As shown in this work by using PEODA instead of PEO the electrolyte can be processed without elevated temperature or the application of processing solvents. Furthermore, the higher Li salt content of P_2_IL_5_S_3_ (30%) compared to PEO_ref_ (18%) enables a higher capacity utilization (short-circuit prevention). Due to the smaller polymer chains P_2_IL_5_S_3_ (3.2 MPa) is more rigid compared to PEO_ref_ (0.3 MPa) providing a more stable film during application.

**Table 2:** Comparison of the developed P_2_IL_5_S_3_ to PEO_ref_ from literature

Name	Polymer type	Composition^a^	Capacity utilization	Processing without temperature or solvents	Elastic modulus
P_2_IL_5_S_3_	Liquid PEODA (*M*_n_: 700)	20 wt% PEODA	8.8 mA h cm^−2^	Yes	3.2 MPa
		50 wt% IL			
		30 wt% Li salt			
PEO_ref_	Solid PEO (*M*_n_: 4 mio)	28 wt% PEO	1.6 mA h cm^−2^	No	0.3 MPa
		54 wt% IL			
		18 wt% Li salt			

a Without BP.

## Conclusion

In this study, a ternary electrolyte (containing polymer, ionic liquid and Li salt) was studied. An alternative electrolyte processing without processing solvents or hot pressing, but taking advantage from a liquid to solid phase transition was enabled. The electrolyte system was sequentially optimized in relation to ionic conductivity and safety. The resulting electrolyte has a high ionic conductivity of >1 mS cm^−1^ at 60 °C and is able to prevent short-circuits due to dendrite growth when containing high salt contents. The electrolyte has a sufficient limiting current density of 0.46 mA cm^−2^ as well as a high thermal stability of >300 °C.

The unique processing at RT and without processing solvents presented in this work paves the way for a new research direction towards ternary electrolytes. Different from gel or solvated processing, the liquid electrolyte can directly be coated and solidified on electrodes allowing for an improved contact towards the electrode surface, thin film applications and fast processing. Also, different coatings for anode and cathode, that allow for electrolyte compositions matching the requirements, could be applied.

## Author contributions

Lukas Herbers^A^ performed the synthesis of electrolytes, cell assembly, electrochemical measurements and evaluation of data. The DSC and TGA measurements were performed by Debbie Berghus^A^. The KF measurements were performed by Lea-Sophie Kemper^A^. Martin Winter^A,B^ and Peter Bieker^B^ created the concept of work and supervised the work. Lukas Herbers^A^ wrote the manuscript through contributions of Verena Küpers^A^, Peter Bieker^B^ and Martin Winter^A,B^.

## Conflicts of interest

There are no conflicts to declare.

## Supplementary Material

RA-013-D3RA02488A-s001
