# Supplementary material for: An ionic liquid- and PEO-based ternary polymer electrolyte for lithium metal batteries: an advanced processing solvent-free approach for solid electrolyte processing
Source: RSC Adv. 2023 Jun 14;13(26):17947–58. doi: 10.1039/d3ra02488a (PMC10265719; doi:10.1039/d3ra02488a)
Supplement: RA-013-D3RA02488A-s001 [file RA-013-D3RA02488A-s001.pdf]

Electronic Supplementary Material (ESI) for RSC Advances.
This journal is © The Royal Society of Chemistry 2023

# Supplementary Information

The PEODA cross-linking follows a three-step radical polymerization.[31,52] First, during UV irradiation benzophenone is excited and radicals are formed by hydrogen abstraction. Second, these radicals react with the double bonds of the monomer PEODA. The formed monomer radical can further react with monomers creating a cross-linked polymer. Third, the radical polymerization is terminated by the formation of a covalent bond of two radicals.

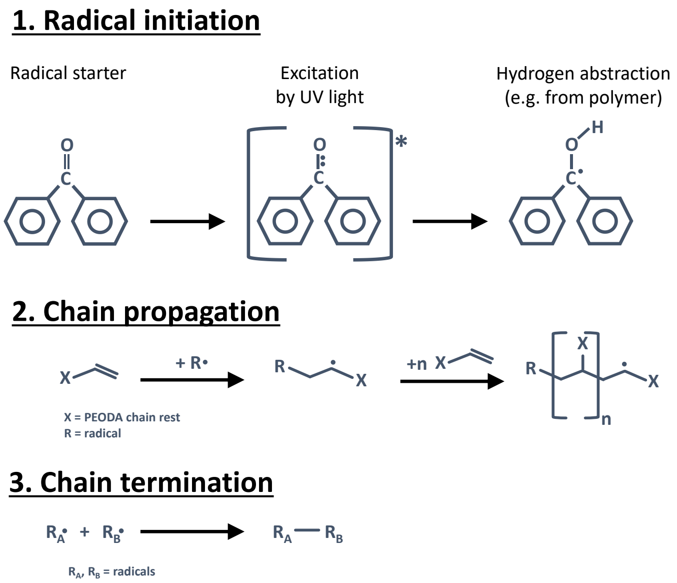


Figure 1. PEODA cross-linking mechanism (radical initiation, chain propagation, chain termination).

The cross-linking process is further evaluated optically and by FT-IR. The *v*(C=C) peak decreases gradually with irradiation time, see Figure 2a. During cross-linking the liquid PEODA turns into a sticky gel after 5 s and becomes a solid polymer film from 15 s, see Figure 2b and c.

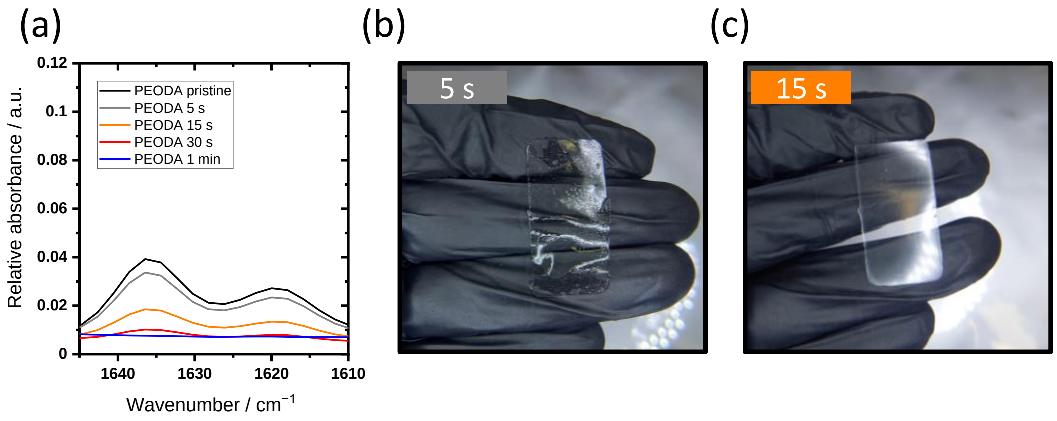


Figure 2. Cross-linking of PEODA. (a) Absorbance spectra of PEODA with 1 wt. % and varying cross-linking duration; (b) photographe of PEODA cross-linked for 5 s; (c) photographe of PEODA cross-linked for 15 s.

The TMA Q400 (TA Instruments) is used to evaluate the elastic modulus of $P_2IL_5L_3$ and $PEO_{ref}$. The tension mode is used with a strain ramp of 3% $min^{-1}$ and the resulting stress is monitored. The elastic modulus is derived from the slope of the stress-strain curve, see Figure 3. The elastic modulus of $P_2IL_5L_3$

is 3.2 MPa, which is higher compared to $PEO_{ref}$ with 0.3 MPa. The higher stiffness of $P_2IL_5L_3$ is a result of the smaller PEO chain length, which decreases the elasticity.

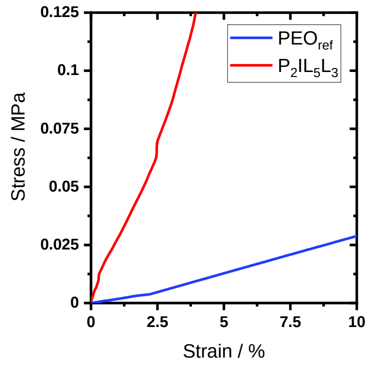


Figure 3 - Stress-strain curve of $P_2IL_5L_3$ and $PEO_{ref}$ measured with a constant strain ramp of 3% $min^{-1}$ at 20 °C.

Differential scanning calorimetry (DSC) analysis was performed with a DSC Q2000 (TA Instruments). A heating rate of 10 K $min^{-1}$ in a temperature range between −100 °C and 100 °C was applied. Hermetic aluminum pans with helium gas flow of 25 mL $min^{-1}$ were used.

A pronounced glass transition is visible at −42 °C to −32 °C for the cross-linked PEODA with 1 wt. % of BP. (Figure 4). This is in agreement of comparable measurements studies in the literature.[53] A glass transition for $P_2IL_5S_3$ could be assumed for the region of −75 °C to −20 °C but the transition is not as pronounced as for PEODA with 1 wt. % BP. No transition can be observed for the region of 0 °C to 100 °C. This agrees with the assumption of a fully amorphous state made earlier.

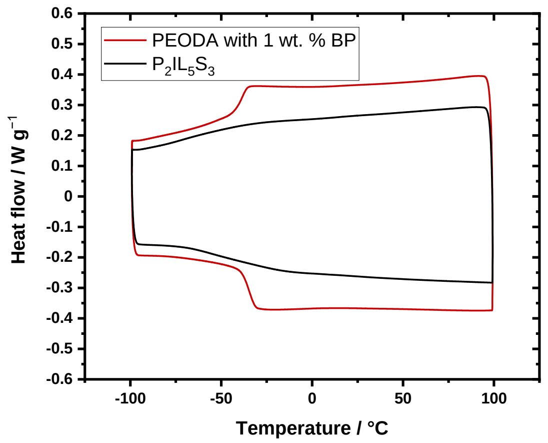


Figure 4. DSC measurements for cross-linked PEODA with 1 wt. % of BP and cross-linked $P_2IL_5S_3$

Curve fits of Arrhenius plots for SST||SST cells with $P_2IL_7S_1$ and $P_2IL_5S_3$ are shown in Figure 5.

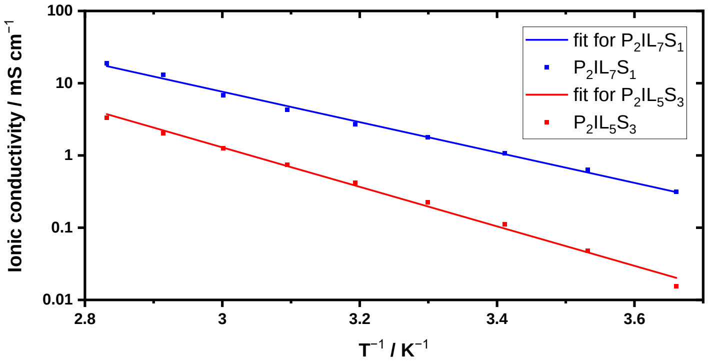


Figure 5. Temperature dependent ionic conductivity of SST||SST coin cells with $P_2IL_7S_1$ (blue dots) and $P_2IL_5S_3$ (red dots). Arrhenius fits $P_2IL_7S_1$ (blue line) and $P_2IL_5S_3$ (red line).

Charge pulse tests were performed in symmetric Li||Li coin cells on a VMP potentiostat (Bio-152 Logic) at 60°C. Charge pulses of ±0.05 mA cm$^{-2}$ to ±0.25 mA cm$^{-2}$ were applied for 0.05 mAh cm$^{-2}$. After each charge pulse the cells were rested till the voltage dropped to 0 V.

The overvoltage development of different electrolyte compositions is studied. (Figure 6) The LiTFSI content increases horizontally from 10 wt. % to 40 wt. % for diagram (a) to (d), respectively (e) to (h). The polymer content is increased vertically from 20 wt. % to 30 wt. % from (a) to (e), (b) to (f), (c) to (g) and (d) to (h). First of all, a clear difference is seen from the upper diagrams (a) to (d) to the lower diagrams (e) to (h). By increasing the polymer fraction by 10 wt. % while keeping the LiTFSI content the same the overvoltages in Li||Li cells increase strongly.

For example, at a current density of 0.05 mA cm$^{-2}$ and a capacity utilization of 0.05 mAh cm$^{-2}$ the overvoltage of Li||Li cells with $P_2IL_7S_1$ is 0.03 V, while the overvoltages of $P_3IL_6S_1$ is 0.08 V. In addition, cells with $P_2IL_7S_1$ reach a plateau in voltage while cells with $P_3IL_6S_1$ do not. When increasing the LiTFSI content from Figure 6 (a) to (d), the overvoltage does not increase notably. For example, at a current density of 0.05 mA cm$^{-2}$ and a capacity utilization of 0.05 mAh cm$^{-2}$ the overvoltage of Li||Li cells with $P_2IL_7S_1$ is 0.03 V while the overvoltage of $P_2IL_4S_4$ cells is 0.05 V. Therefore, increasing the amount of LiTFSI instead of PEODA is beneficial to increase the solid content and reduce the risks of dendrite formation (see **Error! Reference source not found.**).

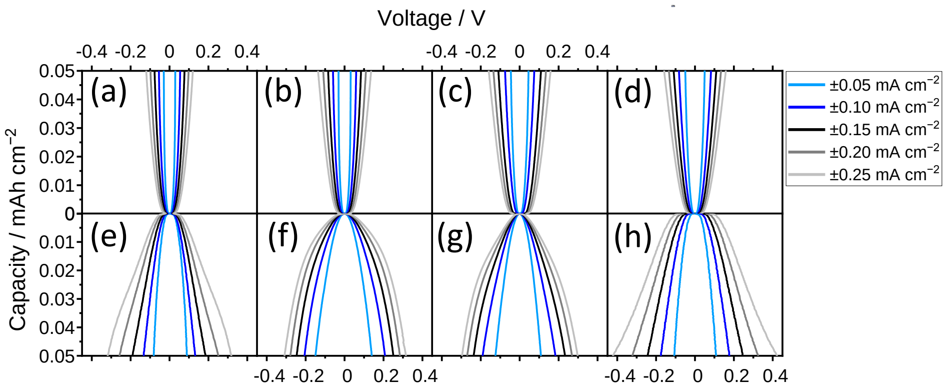


Figure 6. Charge pulse analysis of different electrolyte compositions. (a)$P_2IL_7S_1$; (b)$P_2IL_6S_2$; (c)$P_2IL_5S_3$; (d)$P_2IL_4S_4$; (e)$P_3IL_6S_1$; (f)$P_3IL_5S_2$; (g)$P_3IL_4S_3$; (h)$P_3IL_3S_4$

The morphology of the LFP|$P_2IL_5S_3$|Li cell stack and the Li metal deposition in Cu|$P_2IL_5S_3$|Li cell stacks is evaluated by laser scanning microscopy, see Figure 7 and Figure 8. A 3-D laser scanning microscope (VK-X260 Keyence) was used with a 10x, 20x and 50x magnification lens by Nikon and a 408 nm laser source. In order to visualize the transparent electrolyte combined optical and laser images are shown.

The $P_2IL_5S_3$ electrolyte is capable of contacting both the flat Li metal anode as well as the LFP cathode. The average arithmetic roughness of the electrolyte of 0.9 µm indicates a flat surface. As seen from the magnification of the electrolyte-cathode contact region the rough surface of the LFP cathode is well adhered by the electrolyte.

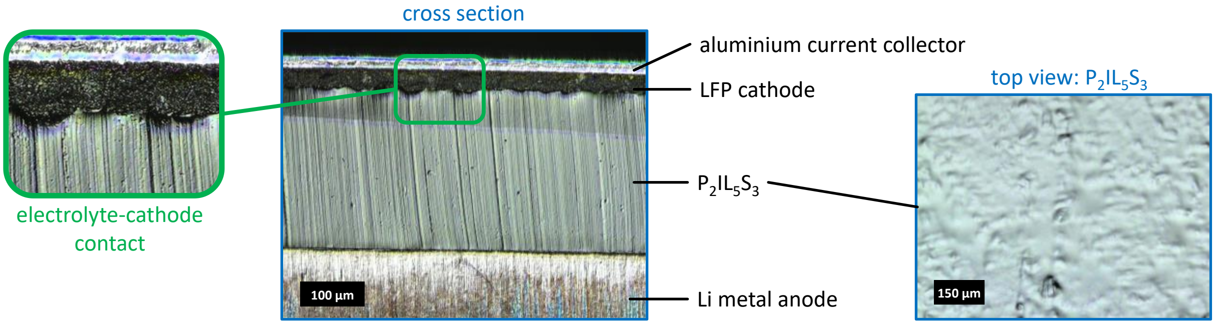


Figure 7. Morphological studies of the LFP|$P_2IL_5S_3$|Li cell stack and the $P_2IL_5S_3$ electrolyte by laser scanning microscopy. Center: combined optical and laser image of the cross section of the LFP|$P_2IL_5S_3$|Li cell stack; left: magnification of the electrolyte-cathode contact; right: top view of a combined optical and laser image of the LFP-facing side of the electrolyte after cell disassembly.

In order to highlight an even and safe Li metal deposition Cu|$P_2IL_5S_3$|Li cell stacks are used. As seen from Figure 8 the Cu surface (Figure 8 a) is evenly covered by Li metal deposits after applying 0.2 mAh $cm^{-2}$ (Figure 8 b). When depositing 2.0 mAh $cm^{-2}$ onto the surface a homogeneous deposition is maintained and the grain size of Li metal deposits is increased, see Figure 8 c. In order to show a uniform Li metal deposition, the soft Li deposits (2.0 mAh $cm^{-2}$) are scratched of the Cu surface using a razor blade, see Figure 8 d. As seen from Figure 8 e an even film of Li metal (yellow and green) is deposited on the Cu surface (blue).

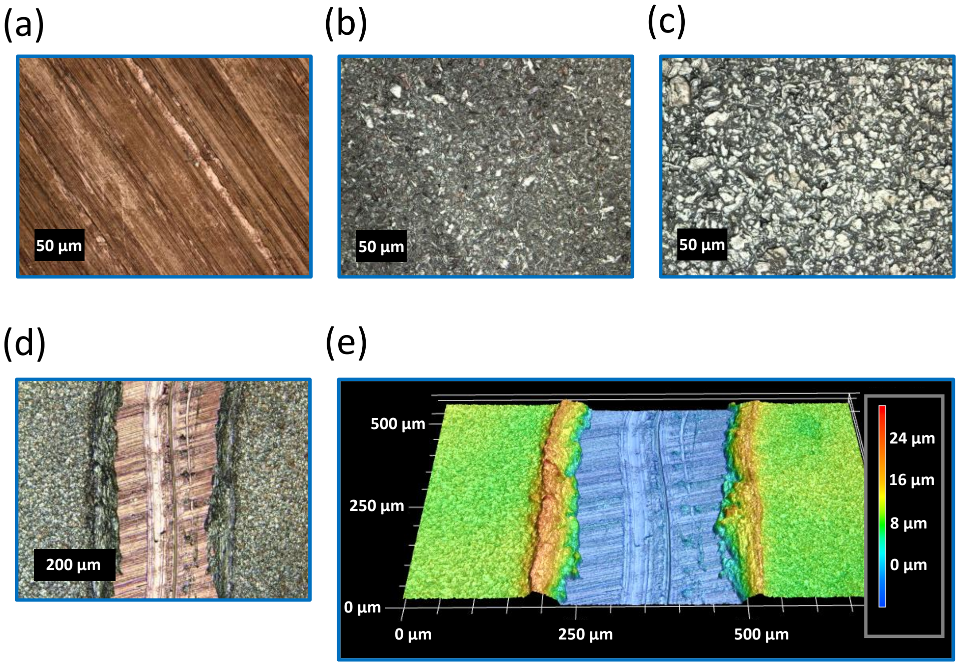


Figure 8. Morphological studies of Li deposition (0.05 mA $cm^{-2}$) in Cu|$P_2IL_5S_3$|Li cell stacks and the $P_2IL_5S_3$ electrolyte by laser scanning microscopy. (a) combined optical and laser image of a pristine Cu electrode surface; (b) combined optical and laser image of a Cu electrode surface covered with Li after deposition of 0.2 mAh $cm^{-2}$; (c) combined optical and laser image of a Cu electrode surface covered with Li after deposition of 2.0 mAh $cm^{-2}$; (c) combined optical and laser image of a Cu electrode surface covered with Li after deposition of 2.0 mAh $cm^{-2}$; (d) image c with the Li deposits partially scratched off; (e) 3D topography of image d.

As shown in Figure 9 $P_2IL_5S_3$ can be cycled in NMC622|$P_2IL_5S_3$|Li cells. Nonetheless, the capacity retention is low (<50 % after 25 cycles). As will be discussed in an upcoming work in order to apply TSPEs a multi-layer approach is necessary. While LFP and Li metal are stable towards $P_2IL_5S_3$, for NMC cathodes the TSPE has to be modified to improve its chemical compatibility towards NMC.

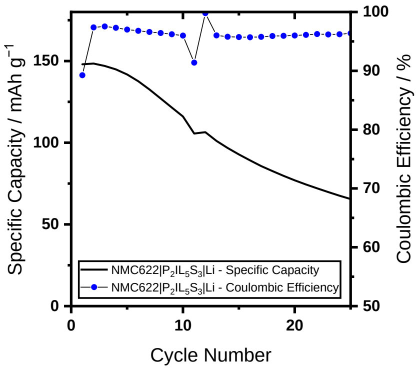


Figure 9. Li metal electrodeposition and –dissolution with a ternary solid electrolyte in NMC622|$P_2IL_5S_3$|Li cells at 0.1 mA $cm^{-2}$ with cut-off voltages from 3.00 V to 4.25 V for 25 cycles at 60 °C. (a) Specific capacity and Coulombic efficiency over cycle number.

## References


31 D. Aidoud, D. Guy-Bouyssou, D. Guyomard, J. Le Bideau and B. Lestriez, Photo-Polymerized Organic Host Network of Ionogels for Lithium Batteries: Effects of Mesh Size and of Ethylene Oxide Content, *ECS Trans.*, 2018, **86**, 163–178.

52 N. D. Carbone, M. Ene, J. R. Lancaster and J. T. Koberstein, Kinetics and Mechanisms of Radical-Based Branching/Cross-Linking Reactions in Preformed Polymers Induced by Benzophenone and Bis-Benzophenone Photoinitiators, *Macromolecules*, 2013, **46**, 5434–5444.

53 T. Keim and K. Gall, Synthesis, characterization, and cyclic stress-influenced degradation of a poly(ethylene glycol)-based poly(beta-amino ester), *J. Biomed. Mater. Res., Part A*, 2010, **92**, 702–711.
